# SP1-induced upregulation of lncRNA SPRY4-IT1 exerts oncogenic properties by scaffolding EZH2/LSD1/DNMT1 and sponging miR-101-3p in cholangiocarcinoma

**DOI:** 10.1186/s13046-018-0747-x

**Published:** 2018-04-11

**Authors:** Yi Xu, Yue Yao, Xingming Jiang, Xiangyu Zhong, Zhidong Wang, Chunlong Li, Pengcheng Kang, Kaiming Leng, Daolin Ji, Zhenglong Li, Lining Huang, Wei Qin, Yunfu Cui

**Affiliations:** 1 Department of Hepatopancreatobiliary Surgery, Second Affiliated Hospital of Harbin Medical University, Harbin, 150086 People’s Republic of China; 2 Department of Endocrinology and Metabolism, Second Affiliated Hospital of Harbin Medical University, Harbin, 150086 People’s Republic of China

**Keywords:** Cholangiocarcinoma, lncRNA, SPRY4-IT1, Scaffold, Oncogenic properties

## Abstract

**Background:**

Accumulating evidence has indicated that long non-coding RNAs (lncRNAs) behave as a novel class of transcription products during multiple cancer processes. However, the mechanisms responsible for their alteration in cholangiocarcinoma (CCA) are not fully understood.

**Methods:**

The expression of SPRY4-IT1 in CCA tissues and cell lines was determined by RT-qPCR, and the association between SPRY4-IT1 transcription and clinicopathologic features was analyzed. Luciferase reporter and chromatin immunoprecipitation (ChIP) assays were performed to explore whether SP1 could bind to the promoter region of SPRY4-IT1 and activate its transcription. The biological function of SPRY4-IT1 in CCA cells was evaluated both in vitro and in vivo. ChIP, RNA binding protein immunoprecipitation (RIP) and luciferase reporter assays were performed to determine the molecular mechanism of SPRY4-IT1 in cell proliferation, apoptosis and invasion.

**Results:**

SPRY4-IT1 was abnormally upregulated in CCA tissues and cells, and this upregulation was correlated with tumor stage and tumor node metastasis (TNM) stage in CCA patients. SPRY4-IT1 overexpression was also an unfavorable prognostic factor for patients with CCA. Additionally, SP1 could bind directly to the SPRY4-IT1 promoter region and activate its transcription. Furthermore, SPRY4-IT1 silencing caused tumor suppressive effects via reducing cell proliferation, migration and invasion; inducing cell apoptosis and reversing the epithelial-to-mesenchymal transition (EMT) process in CCA cells. Mechanistically, enhancer of zeste homolog 2 (EZH2) along with the lysine specific demethylase 1 (LSD1) or DNA methyltransferase 1 (DNMT1) were recruited by SPRY4-IT1, which functioned as a scaffold. Importantly, SPRY4-IT1 positively regulated the expression of EZH2 through sponging miR-101-3p.

**Conclusions:**

Our data illustrate how SPRY4-IT1 plays an oncogenic role in CCA and may offer a potential therapeutic target for treating CCA.

**Electronic supplementary material:**

The online version of this article (10.1186/s13046-018-0747-x) contains supplementary material, which is available to authorized users.

## Background

Cholangiocarcinoma (CCA) is a highly aggressive neoplasm that originates from cholangiocytes and has increasing incidence and prevalence rates [1]. Currently, there is no effective chemoprevention or radiotherapy for CCA [2]. Radical resection offers the only curative option, but it is suitable for only a minority of patients who are diagnosed at the early stages of the disease [3]. What is worse, despite advances in surgical techniques and an improved understanding of the role of vascular resection and reconstruction, the 5-year survival rates after radical surgery range from 15% to 35% for CCA [4]. For the majority of patients with unresectable tumors, the median overall survival is often less than 12 months with palliative treatment [5]. CCA is a complex biological process that results from the dysregulation of many cancer-related genes. Therefore, an improved understanding of the molecular mechanisms underlying the pathogenesis of CCA will supply an arm for building up effective diagnostic and therapeutic targets for CCA.

Long non-coding RNAs (lncRNAs) are a class of transcribed RNA molecules longer than 200 nucleotides that have no significant protein-coding potential [6–8]. LncRNAs are involved in various biological processes, such as nuclear import, nuclear and cytoplasmic trafficking, alternative splicing and cell cycle arrest [9, 10]. A number of studies have demonstrated that the aberrant expression of lncRNAs could be critical in understanding the harmful effects of cancer on regulating cellular processes, including proliferation, metastasis and differentiation [11, 12]. Recently, several studies have demonstrated that lncRNAs can function as a molecular scaffold that interacts with proteins or RNAs to indirectly exert biological functions. For instance, enhancer of zeste homolog 2 (EZH2), a subunit of polycomb repressive complex 2 (PRC2), is a methyltransferase for histone H3 lysine 27 trimethylation (H3K27me3) [13]. Chen et al. reported that lincRNA 00152 is involved in the transcriptional repression of IL24 and facilitates lung adenocarcinoma proliferation via binding to EZH2 [14]. Additionally, lncRNAs can also affect post-transcriptional processing by functioning as a ‘sponge’ to absorb miRNAs. A recent study showed that MALAT1 could serve as a competing endogenous RNA (ceRNA) by sponging miR-23b-3p in gastric cancer [15]. However, to the best of our knowledge, research regarding the involvement of lncRNAs in CCA has just begun.

SPRY4-intronic transcript 1 (SPRY4-IT1) is located at 5q31.3 and derived from an intron region within the SPRY4 gene. SPRY4-IT1 was first identified as an oncogene in melanoma cells, in which it plays a vital role in cell proliferation, apoptosis, migration and invasion [16]. Conversely, Sun et al. found that downregulated SPRY-IT1 facilitates non-small-cell lung cancer cell proliferation and metastasis by affecting epithelial–mesenchymal transition [17]. Liu et al. reported that SPRY4-IT1 is significantly upregulated in bladder cancer and exerts its oncogenic function by sponging miR-101-3p [18]. Also, SPRY4-IT1 increases the proliferation of breast cancer cells by upregulating ZNF703 expression proved by a recent study [19]. However, the expression profile, clinical features, functional role and underlying targets of SPRY4-IT1-related carcinogenesis in CCA have not been characterized.

In this study, we designed experiments to explore the expression pattern, functional role and underlying mechanisms of SPRY4-IT1 in CCA. Cell proliferation, apoptosis, migration, invasion and epithelial-to-mesenchymal transition (EMT) after SPRY4-IT1 silencing were assessed, which implied that SPRY4-IT1 might exert oncogenic properties in CCA. Furthermore, SPRY4-IT1 transcription could be activated by transcription factor SP1. More importantly, SPRY4-IT1 interacts with EZH2, lysine specific demethylase 1 (LSD1) and DNA methyltransferase 1 (DNMT1) and may function as a scaffold for them, thereby suppressing the underlying targets KLF2 and LATS2. SPRY4-IT1 could also positively elevate EZH2 expression levels through sponging miR-101-3p at posttranscriptional level.

## Methods

### Clinical specimens and cell lines

Seventy CCA tissue samples and their paired adjacent non-tumorous bile duct tissues were acquired from patients who underwent radical surgery at the Second Affiliated Hospital of Harbin Medical University between 2010 and 2012 with informed consent. This study was carried out with the approval of the Ethics Review Committees of Harbin Medical University. No local or systemic treatments were administered to these patients before surgery. The criteria for sample collection: tumor tissues contained at least 70% tumor cells, while the matched non-cancerous tissues contained no tumor cells. Complete clinic-pathological follow-up data for the patients were collected. RBE and HCCC-9810 cells were obtained commercially from the Cell Bank of Type Culture of the Chinese Academy of Sciences (Shanghai, China). Human intrahepatic biliary epithelial cell (HIBEC) and other CCA cells (CCLP-1, HuCCT1, Huh-28, KMBC and QBC939) were preserved in our laboratory. Cells were maintained in RPMI-1640 (Gibco, Grand Island, NY, USA) containing 10% fetal bovine serum (Invitrogen Life Technologies, Carlsbad, CA, USA) in a humidified atmosphere at 37 °C and 5% CO_2_. All cell lines were passaged for no more than 6 months.

### Cell transfection and RT-qPCR analysis

Short-hairpin RNA oligos directed against SPRY4-IT1 were synthesized with each containing 4 nucleotide overhangs necessary for directional cloning, Then, oligos were annealed and ligated into the shRNA vector (GeneChem, Shanghai, China). All siRNA and shRNA sequences are listed in Additional file 1: Table S1. To ectopic the expression of SP1/SPRY4-IT1/KLF2/LATS2 in CCA cells, the expression plasmid for SP1 (NM_138473), SPRY4-IT1 (NR_131221), KLF2 (NM_016270), and LATS2 (NM_014572) were PCR-amplified and subcloned into the XhoI and KpnI sites of the pcDNA3.1 vector, respectively (GeneChem, Shanghai, China). An empty pcDNA 3.1 vector was used as a control. Plasmid vectors for transfection were prepared using DNA Miniprep or Midiprep kits (Qiagen, Hilden, Germany), and transfected into cells using Lipofectamine 3000 (Thermo Fisher Scientific, USA). At 48 h post-transfection, cells were harvested for further analyses. Total RNA was isolated from tissue samples or cells using Trizol (Thermo Fisher Scientific, Waltham, MA, USA). RNA integrity number (RIN) analysis was performed using an Agilent 2100 Bioanalyser and RNA 6000 LabChip kit with Agilent 2100 Expert software (Agilent Technologies). The isolated RNA from tissue samples with a RIN of 7 or more was considered usable in the study. The Transcriptor First Strand cDNA Synthesis Kit (Roche, Germany) was used to reverse-transcribe RNA into complementary DNA. RT-qPCR was carried out using the FastStart Universal SYBR Green Master Kit (Roche, Germany) and a BIO-RAD C1000 Thermal Cycler according to the manufacturer’s instructions. All primer sequences are summarized in Additional file 1: Table S1. We constructed standard curves prior to quantification of relative gene expression. Amplification efficiencies (E) were calculated from the slope of the standard curves according to the equation: E = 10 ^[− 1/slope]^ -1, and they ranged from 90% to 100%. The fold change of the relative expression was calculated by using the delta-delta CT method.

### Cell viability and colony formation assays

Cell counting kit-8 (CCK-8) and Ki67 immunofluorescence staining assays were used to assess cell viability. Cell proliferation was documented following the manufacturer’s directions of CCK-8 kit (Dojindo, Japan) every 24 h. For the Ki67 immunofluorescence staining, transfected cells were placed in 24-well plates on cover glasses. Briefly, cells were fixed in immunostaining permeabilization buffer with Triton X-100 (Beyotime, Beijing, China) and then blocked with 5% bovine serum albumin. After the cells were incubated with anti-Ki67 monoclonal antibody (Abcam, Cambridge, MA, USA) and probed with green fluorescent-labeled secondary antibody (Solarbio, Beijing, China), an inverted fluorescence microscope (Leica, Germany) was used to observe the cells. For the clonogenic assays, a particular number of transfected cells were resuspended in a single-cell suspension and plated in six-well dishes; then, the cells were cultivated with RPMI-1640 containing 10% FBS for approximately 2 weeks until visible colonies formed. The colonies were fixed in paraformaldehyde and stained with crystal violet solution (Beyotime, Beijing, China) before observation.

### Flow cytometry analyses for cell apoptosis and TdT-mediated dUTP Nick-end labeling (TUNEL) assay

Cells transfected with the indicated siRNAs and in the exponential growth phase were detached by trypsinization and resuspended in Annexin V-FITC binding buffer. Afterwards, Annexin V and propidium iodide were added to the cell suspension and incubated for 15 min at room temperature. After that, the stained cells were analyzed by using flow cytometry (FACScan, BD Biosciences). For TUNEL assay, after the cells were fixed in paraformaldehyde and treated with immunostaining permeabilization buffer with Triton X-100 (Beyotime, Beijing, China), they were incubated with 45 μl of fluorescent-labeled reagent and 5 μl of terminal deoxynucleotidyl transferase (Beyotime, Beijing, China) at 37 °C for 1 h. 4′,6-diamidino-2-phenylindole (DAPI) was used to color nuclear fractions. Finally, the cells were observed and photographed using a fluorescence microscope (Leica, Germany).

### Caspase-3/9 activity assay

A caspase-3/9 activity kit (Solarbio, Beijing, China) was used for determination of caspase-3/9 activity in cell lysates following the manufacturer’s protocol.

### Cell migration and invasion assays

Cell motility was measured by scratch wound assays. A 200 μL pipette tube was used to create a scraped, acellular area. Photographs were taken after 0 h and 36 h to evaluate the motility of each transfected group. Migration was quantified by counting the average distance that cells migrated towards the original wound field. Transwell migration and invasion assays were performed as previously described [20, 21]. In brief, cells were placed in serum-free RPMI-1640 medium in the upper chambers of transwell units (Costar, Washington, DC, USA) with or without 40 μL of Matrigel (BD Biosciences) coating. The lower compartments were filled with complete medium. After 24 h of incubation, cells in the upper membrane were removed with a cotton swab. The cells that traversed the membrane were fixed in 4% paraformaldehyde and stained with crystal violet solution.

### Tumor xenograft study

The animal experiments were approved and reviewed by the Animal Care and Use Committee of Harbin Medical University. HuCCT1 cells were transfected with shSPRY4-IT1 or the scrambled control. After 48 h, 3 × 10^6^ cells were injected subcutaneously into either side of the posterior flank of female BALB/c nude mice (6 weeks of age, *n* = 6 per group). Tumor growth was measured every 3 days, and tumor volumes were calculated using the following equation: V = 0.5 × D × d^2^ (V, volume; D, longitudinal diameter; d, latitudinal diameter). The mice were euthanized 18 days after injection, and the tumors weights were measured. The location and relative expression level of Ki67 was measured by the avidin-biotin-peroxidase method. In addition, total RNA was extracted from the tumors to measure the expression levels of SPRY4-IT1.

### Subcellular fractionation

Nuclear and cytosolic fraction separation was performed using a PARIS kit (Life Technologies, Shanghai, China), according to the manufacturer’s directions.

### Luciferase reporter assay

The SP1 binding motif in the promoter region of SPRY4-IT1 was identified by JASPAR (http://jaspar.genereg.net/). The different fragment sequences were synthesized and inserted into a pGL3-basic vector (Promega, Madison, WI). All vectors were verified by sequencing and luciferase activities were assessed using the Dual Luciferase Assay Kit (Promega).

### RNA immunoprecipitation (RIP) assay

RIP was carried out by using the EZMagna RIP kit (Millipore, Billerica, MA, USA) according to the manufacturer’s instructions. In brief, HuCCT1 and RBE cells were lysed in RIP lysis buffer; then, the lysates were incubated with magnetic beads conjugated with specific antibodies or control IgG (Millipore). Afterwards, the beads were washed and incubated with Proteinase K to remove the proteins. At last, purified RNA was subjected to RT-qPCR analysis.

### Chromatin immunoprecipitation (ChIP) assay

ChIP assays were performed by using the Magna ChIP Kit (Millipore, Bedford, MA) in accordance with the manufacturer’s directions. HuCCT1 and RBE cells were treated with formaldehyde to generate DNA-protein cross-links. Cell lysates were sonicated to generate chromatin fragments of 200–300 bp, and the lysates were immunoprecipitated with specific antibodies or IgG as the control. The precipitated chromatin DNA was recovered and measured by qPCR.

### Western blotting and immunohistochemistry (IHC) assays

Western blot and IHC analysis were carried out following standard protocols as previously described [21].

### Statistical analysis

Statistical analyses were performed by using GraphPad Prism 5.01 software (GraphPad Software, Inc., La Jolla, CA, USA) and SPSS 19.0 statistical software package (IBM, Armonk, NY, USA). Significant differences between groups were evaluated by a paired, two-tailed Student’s *t*-tests. The results are reported as the means ± standard deviation (SD) based on at least three replicates. The relationship between SPRY4-IT1 levels and clinicopathological features was calculated by Fisher’s exact tests. Survival curves were drawn by Kaplan-Meier analyses and tested using log-rank tests. Survival data were analyzed by using univariate and multivariate Cox proportional hazards modeling. Statistical significance was set at *P* < 0.05.

## Results

### The transcription of SPRY4-IT1 is specifically up-regulated in CCA and is related to aggressive tumor phenotypes and unfavorable prognosis

**Fig. 1:**
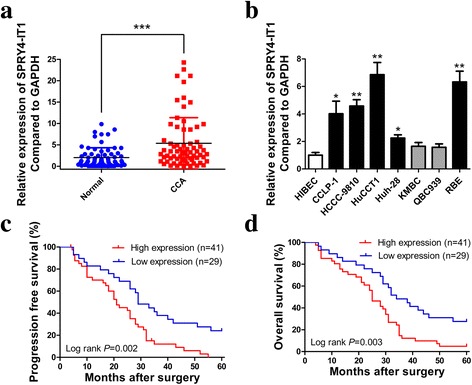
SPRY4-IT1 is highly expressed in CCA tissues/cells and associated with poor prognosis of CCA. **a** Relative expression of SPRY4-IT1 in 70 CCA tissue samples and their paired non-cancerous tissue samples measured by RT-qPCR. **b** Relative expression of SPRY4-IT1 in HIBEC and CCA cell lines measured by RT-qPCR. **c**-**d** Kaplan-Meier PFS and OS analyses of the association between SPRY4-IT1 expression and CCA patient survival ability. *P < 0.05, **P < 0.01, ***P < 0.001

RT-qPCR was used to measure the SPRY4-IT1 expression levels in a cohort of 70 CCA tumors and their paired adjacent non-tumorous samples. The results demonstrated that SPRY4-IT1 was dramatically increased in CCA samples compared with those in the normal counterparts (Fig. 1a). To further investigate the function of SPRY4-IT1 in CCA, a panel of human CCA cell lines was evaluated by RT-qPCR. As shown in Fig. 1b, SPRY4-IT1 was present at higher levels in CCA cells (CCLP-1, HCCC-9810, HuCCT1, Huh-28 and RBE) compared with HIBEC. To further investigate the clinical significance of abnormal SPRY4-IT1 transcript levels in CCA patients, correlations between SPRY4-IT1 levels and clinicopathological characteristics were explored. RT-qPCR analysis determined that the expression level of SPRY4-IT1 was 2.65-fold higher in the CCA samples than the paired normal biliary tissues. Thus, the 70 CCA patients were classified into low (< the average value) or high (> the average value) expression groups. The results calculated by Fisher’s exact tests revealed that SPRY4-IT1 expression was predominantly increased in late tumor stage (*P* = 0.013) and positively associated with advanced tumor node metastasis (TNM) stage (*P* = 0.038). However, no correlation between SPRY4-IT1 expression and other parameters were found (Additional file 2: Table S2). Kaplan-Meier analyses and log-rank tests showed that higher SPRY4-IT1 expression levels indicated worse progression free survival (PFS) and overall survival (OS) after radical surgery in patients with CCA (Fig. 1c and d). To further validate the prognostic value of SPRY4-IT1 in CCA patients, we performed univariate and multivariate Cox regression analyses. The data confirmed that SPRY4-IT1 expression could be regarded as an independent predictor of an adverse PFS (*P* = 0.018) and OS (*P* = 0.027) in CCA patients, in addition to TNM stage (*P* = 0.005, Additional file 3: Table S3).

### SP1 activates SPRY4-IT1 transcription in CCA cells

**Fig. 2:**
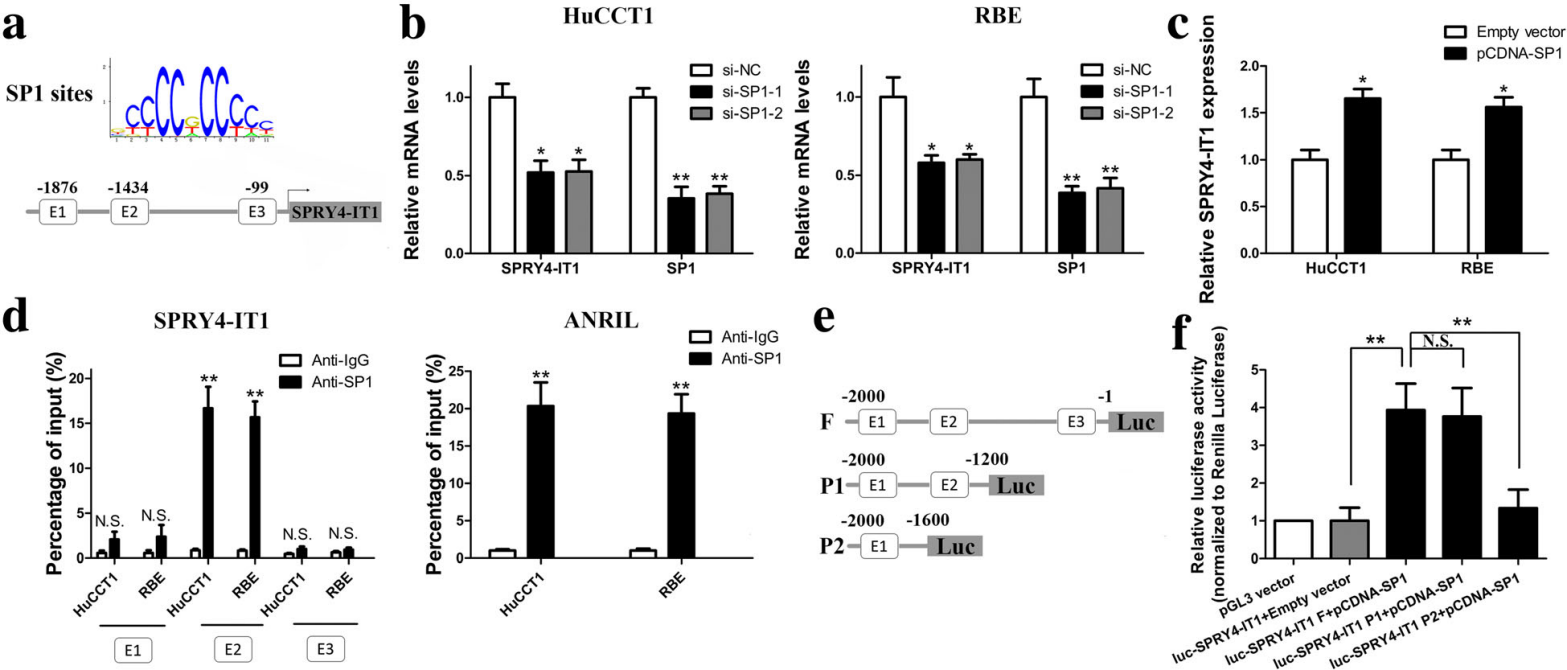
SP1 binds to the promoter region of SPRY4-IT1 and activates its transcription. **a** SP1 binding site prediction in the SPRY4-IT1 promoter region using JASPAR. **b** RT-qPCR analysis of SPRY4-IT1 and SP1 expression in HuCCT1 and RBE cells after SP1 silencing. **c** RT-qPCR analysis of SPRY4-IT1 expression in HuCCT1 and RBE cells after SP1 overexpression. **d** ChIP-qPCR analysis of SP1 occupancy in the SPRY4-IT1 promoter in HuCCT1 and RBE cells. ANRIL was used as a positive control and IgG as a negative control. **e** Construction of the luciferase reporter vector. **f** Dual luciferase reporter assays were used to determine the SP1 binding site on the SPRY4-IT1 promoter region. *P < 0.05, **P < 0.01

Increasing evidence indicated that transcription factors such as E2F1, SP1 could activate the transcription of downstream targets including lncRNAs [22]. JASPAR online database was used to predict the potential transcription factor and SP1 is predicted to be bound to the SPRY4-IT1 promoter region with high scores (Fig. 2a and Additional file 4: Figure S1A). After silencing of SP1 by siRNAs, the expression of SPRY4-IT1 was decreased (Fig. 2b). Moreover, SP1 overexpression facilitated SPRY4-IT1 expression (Fig. 2c and Additional file 4: Figure S1B). Subsequent ChIP assays documented an obvious SP1-binding activity on the endogenous SPRY4-IT1 promoter region around E2. ANRIL, a known SP1 target, was used as a positive control. (Fig. 2d). Additionally, luciferase reporter assays indicated that SP1 binds to the E2 (− 1434 bp) binding site, but not the other two sites (Fig. 2e and f).

### Knockdown of SPRY4-IT1 inhibits cell growth in vitro and in vivo

**Fig. 3:**
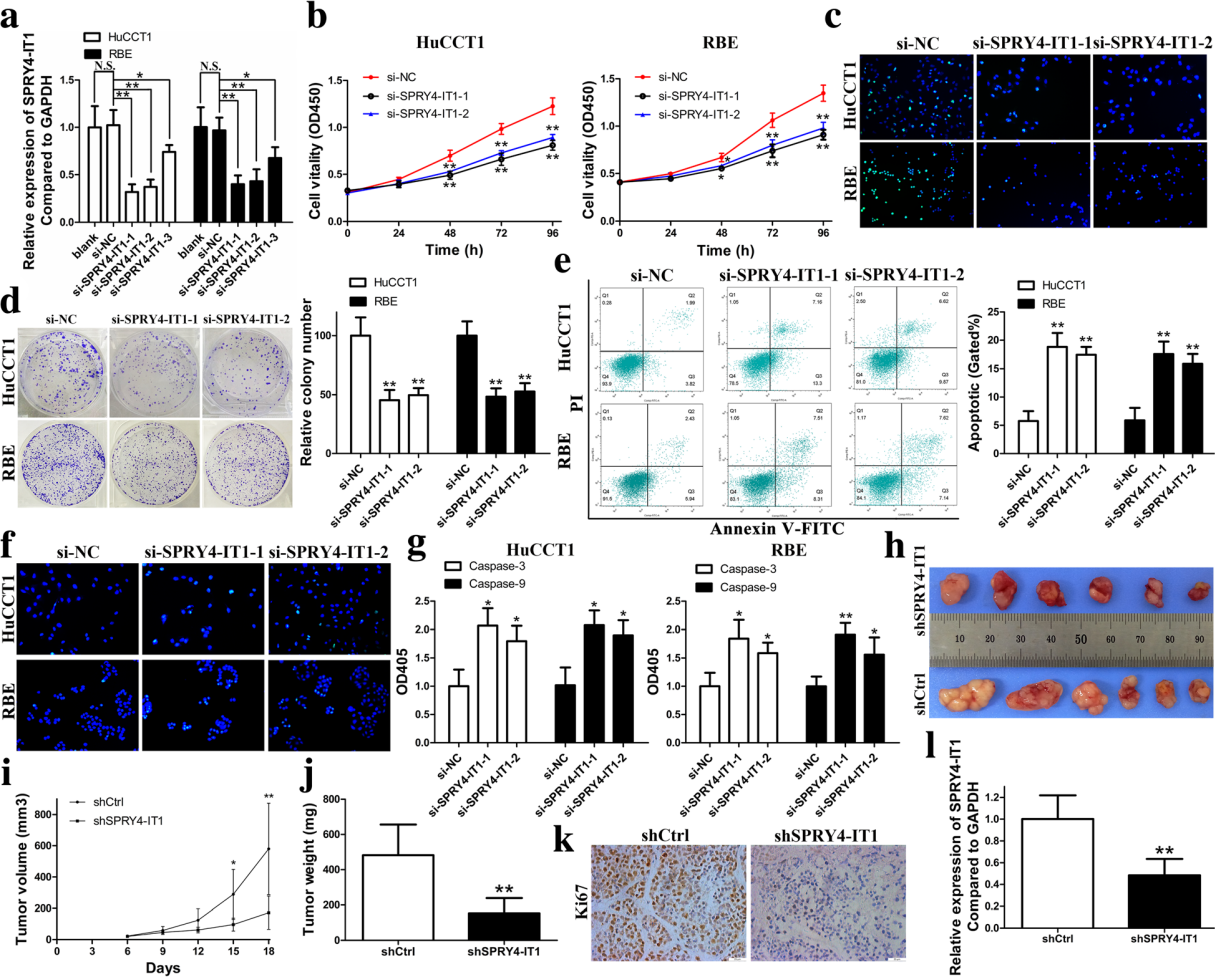
SPRY4-IT1 knockdown inhibits CCA cell growth in vitro and in vivo. **a** Relative expression of SPRY4-IT1 in HuCCT1 and RBE cells measured by RT-qPCR. **b** Proliferation curves were determined in HuCCT1 and RBE cells after transfected with si-SPRY4-IT1–1, si-SPRY4-IT1–2 or si-NC by CCK-8 assays. **c** The location and relative expression of Ki67 were detected in HuCCT1 and RBE cells after transfected with si-SPRY4-IT1–1, si-SPRY4-IT1–2 or si-NC by Ki67 immunofluorescent staining assays. Ki67 positive cells were labeled with green fluorescence; nuclear fractions were labeled with DAPI (blue). **d** Colony-forming abilities were measured in HuCCT1 and RBE cells after transfected with si-SPRY4-IT1–1, si-SPRY4-IT1–2 or si-NC by clonogenic assays. **e** Cell apoptosis was detected in HuCCT1 and RBE cells after transfected with si-SPRY4-IT1–1, si-SPRY4-IT1–2 or si-NC by flow cytometry. **f** Cell apoptosis was detected in HuCCT1 and RBE cells after transfected with si-SPRY4-IT1–1, si-SPRY4-IT1–2 or si-NC by TUNEL staining assays. Apoptotic cells were labeled with TUNEL (green); nuclear fractions were labeled with DAPI (blue). **g** Relative expression of caspase-3 and caspase-9 were detected in HuCCT1 and RBE cells after transfected with si-SPRY4-IT1–1, si-SPRY4-IT1–2 or si-NC by caspase-3/9 activity assays. **h** 18 days after injection of HuCCT1 cells transfected with shSPRY4-IT1 or shCtrl, tumors were removed from nude mice. **i** Tumor volumes were measured every 3 days after injection. **j** Tumor weights were measured after the tumors were harvested. **k** The tumor sections were under IHC staining using antibodies against Ki67. **l** RT-qPCR was conducted to determine the average expression of SPRY4-IT1 in xenograft tumors. *P < 0.05, **P < 0.01

**Fig. 4:**
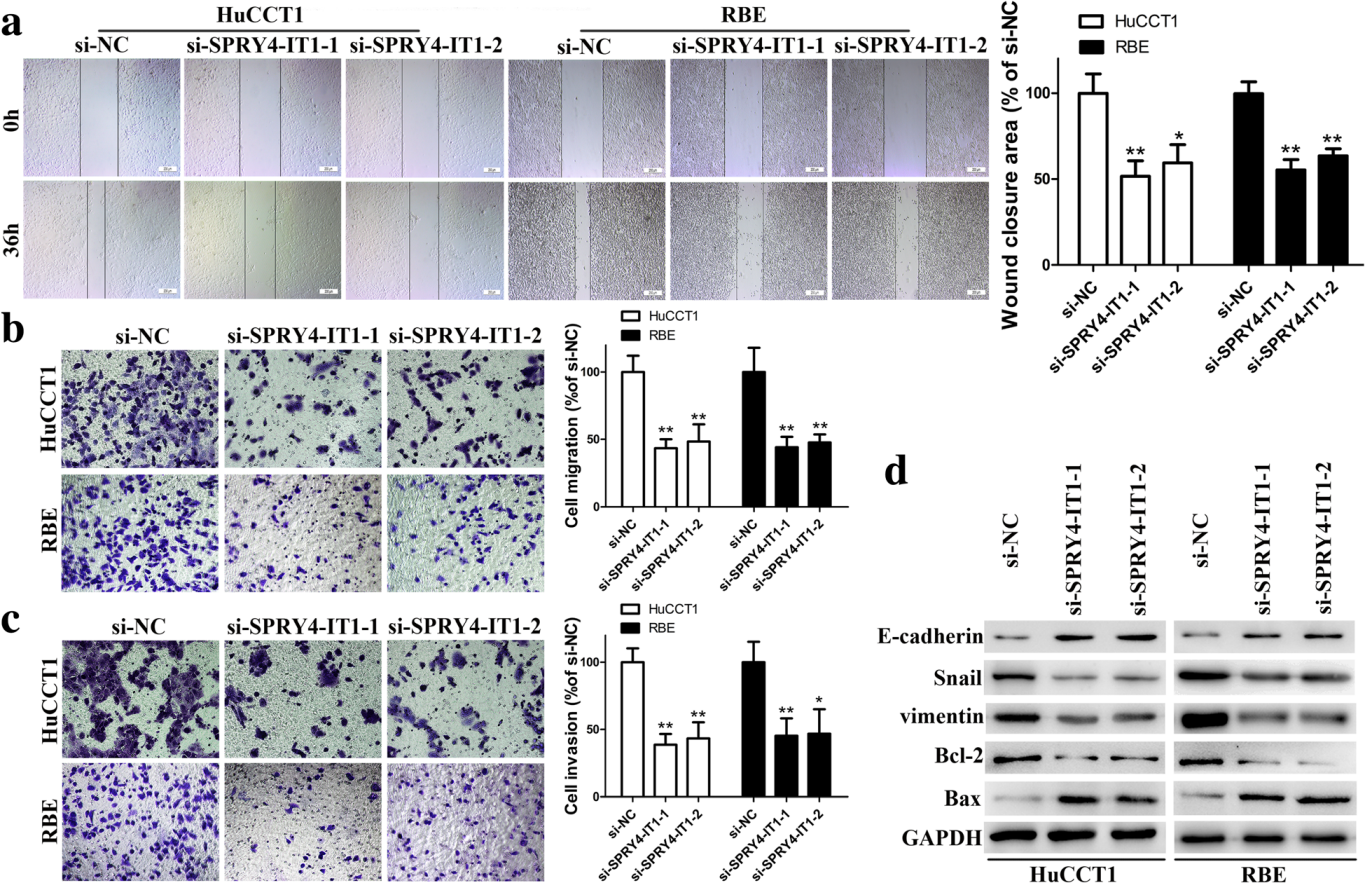
SPRY4-IT1 promotes cell metastatic properties by reversing EMT. **a** The migration capacities were detected in HuCCT1 and RBE cells transfected with si-SPRY4-IT1–1, si-SPRY4-IT1–2 or si-NC by wound healing assays. **b**-**c** The migration and invasive capacities were detected in HuCCT1 and RBE cells transfected with si-SPRY4-IT1–1, si-SPRY4-IT1–2 or si-NC by transwell assays. **d** E-cadherin, Snail, vimentin, Bcl-2 and Bax protein levels were detected in HuCCT1 and RBE cells transfected with si-SPRY4-IT1–1, si-SPRY4-IT1–2 or si-NC by western blotting. *P < 0.05, **P < 0.01

Given that SPRY4-IT1 is upregulated in CCA tissues and cell lines, it is necessary to determine whether SPRY4-IT1 suppression could affect biologic activity in CCA cells. To achieve this, we performed loss-of-function studies in HuCCT1 and RBE cells that with higher SPRY4-IT1 expression. Three siRNAs targeting SPRY4-IT1 were designed to suppress SPRY4-IT1 expression. At 48 h post-transfection, RT-qPCR data indicated that all the three siRNAs caused a significant loss of SPRY4-IT1 expression, the most efficient two of which (si-SPRY4-IT1–1 and si-SPRY4-IT1–2) were selected for the subsequent in vitro experiments (Fig. 3a). The proliferation curves determined by CCK-8 assays demonstrated that cell growth was remarkedly attenuated by SPRY4-IT1 knockdown in HuCCT1 and RBE cells (Fig. 3b). Expression of Ki67 is correlated with the proliferative activity of intrinsic cell populations in malignancies, allowing it to be used as a marker of tumor cell aggressiveness. In this study, Ki67 immunofluorescence assays indicated that SPRY4-IT1 inhibition suppressed proliferation in CCA cells compared with negative control (Fig. 3c). Additionally, SPRY4-IT1 knockdown significantly impaired clonogenic ability in CCA cells (Fig. 3d). Furthermore, the proportion of apoptotic cells after SPRY4-IT1 silencing was dramatically increased in HuCCT1 and RBE cells as shown by flow cytometry assays (Fig. 3e). Subsequent TUNEL staining assays revealed similar results (Fig. 3f). The activities of caspase-3 and caspase-9 were both higher in the si-SPRY4-IT1–1 and − 2 groups than the negative control (Fig. 3g). Western blotting results showed that silencing SPRY4-IT1 could inhibit Bcl-2 expression and increase Bax protein levels compared to those in the si-NC groups (Fig. 4d). To further validate whether SPRY4-IT1 expression can influence tumorigenesis in vivo, we injected HuCCT1 cells transfected with shSPRY4-IT1/shCtrl into BALB/c nude mice. In accordance with previous in vitro results, tumor growth was dramatically slower in the shSPRY4-IT1 group than the shCtrl group (Fig. 3h and i). In addition, 18 days after inoculation, tumor weights were markedly lower in the shSPRY4-IT1 group than the shCtrl group (Fig. 3j). What’s more, tumors formed from shSPRY4-IT1-transfected cells had lower Ki67 expression levels than tumors formed from shCtrl cells as determined by IHC assays (Fig. 3k). The RT-qPCR data revealed lower expression levels of SPRY4-IT1 in the tumors formed from shSPRY4-IT1 group (Fig. 3l).

### Inhibiting SPRY4-IT1 expression impairs metastatic properties by reversing EMT in CCA cells

We further explored the potential impact of SPRY4-IT1 on metastatic properties in CCA cells by using wound healing and transwell assays. Although HuCCT1 and RBE cells transfected with si-NC showed robust in vitro migration capabilities, silencing SPRY4-IT1 with either of the two siRNAs dramatically impaired the wound closure potential (Fig. 4a). Consistent with our predictions, transwell experiments revealed significantly decreased migration and invasive capabilities in the si-SPRY4-IT1–1 and si-SPRY4-IT1–2 groups (Fig. 4b and c). Cell migration and invasion are essential prerequisites for cancer metastasis, and EMT is a pivotal mechanism that is correlated with cell invasive capabilities. To assess whether SPRY4-IT1 was also involved in the EMT in CCA, the expression levels of EMT-related proteins were evaluated in HuCCT1 and RBE cells after transfected with the indicated siRNAs. Western blot analyses showed that silencing SPRY4-IT1 increased the expression levels of E-cadherin and decreased the expression levels of Snail and vimentin (Fig. 4d).

### SPRY4-IT1 represses KLF2 and LATS2 expression by scaffolding EZH2, LSD1 and DNMT1

**Fig. 5:**
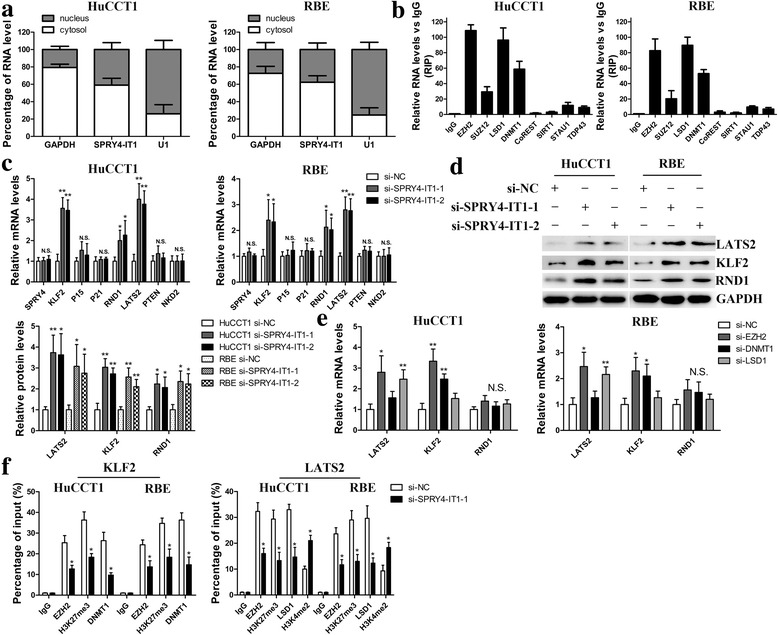
SPRY4-IT1 functions as a scaffold for PRC2/LSD1/DNMT1 to regulate KLF2 and LATS2 expression in CCA cells. **a** RT-qPCR detection of the percentage of SPRY4-IT1, GAPDH and U1 in the cytoplasm and nuclear fractions of HuCCT1 and RBE cells. GAPDH and U1 were used as cytoplasmic and nuclear localization markers, respectively. **b** SPRY4-IT1 levels in immunoprecipitates were determined by RT-qPCR. SPRY4-IT1 expression levels are presented as fold enrichment values relative to IgG immunoprecipitates. **c** RT-qPCR was performed to confirm the selected gene expression in SPRY4-IT1 knockdown cells. **d** Western blot assays were performed to confirm the selected gene expression in SPRY4-IT1 knockdown cells. **e** KLF2 and LATS2 expression levels were examined in cells transfected with EZH2, LSD1 or DNMT1 siRNAs. **f** ChIP-qPCR analysis of EZH2, H3K27me3, LSD1, H3K4me2 and DNMT1 occupancy on the KLF2 and LATS2 promoter region in HuCCT1 and RBE cells after transfected with si-SPRY4-IT1–1 or si-NC. *P < 0.05, **P < 0.01

Commonly, lncRNAs modulate their downstream target genes via binding to RNA binding proteins or functioning as endogenous competing RNAs for miRNAs. To identify the potential mechanism underlying SPRY4-IT1-associated regulation of biological features in CCA cells, subcellular fractionation analysis was first performed. The results demonstrated that SPRY4-IT1 is distributed in both the cytoplasm and nucleus, and the ratio of SPRY4-IT1 in the cytoplasm is higher than nucleus (about 60% vs. 40%) (Fig. 5a). Thus, we inferred that SPRY4-IT1 might regulate downstream targets at both transcriptional and post-transcriptional levels. Afterwards, the binding probabilities between SPRY4-IT1 and RNA binding proteins were analyzed by RNA-protein interaction prediction (http://pridb.gdcb.iastate.edu/RPISeq/). The data showed that SPRY4-IT1 potentially binds EZH2, SUZ12, LSD1, DNMT1, CoREST, SIRT1, STAU1 and TDP43 (with RF and SVM scores > 0.5, Additional file 5: Figure S2A). RIP assays were conducted to analyze the interaction of SPRY4-IT1 and these RNA binding proteins and the results showed a strong physical interaction between SPRY4-IT1 and EZH2, LSD1 and DNMT1, while U1 binding with SNRNP70 was used as a positive control (Fig. 5b and Additional file 5: Figure S2B). EZH2, LSD1 and DNMT1 are histone/DNA methylation regulators distributed mainly in nuclear fractions. Thus, the findings above suggested that SPRY4-IT1 might epigenetically repress underlying targets expression at transcriptional level. Next, we selected several EZH2, LSD1 or DNMT1 potential targets (P15, P21, LATS2, RND1, KLF2, NKD2, PTEN) with tumor-suppressor function and SPRY4, and hypothesized that they may involve in the contributions of SPRY4-IT1 to CCA progression. The RT-qPCR data showed that attenuation of SPRY4-IT1 expression led to increased KLF2, RND1 and LATS2 expression, whereas in the other genes, there was no significant difference (Fig. 5c). Furthermore, Western blot analysis showed the same results (Fig. 5d), which indicated that KLF2, RND1 and LATS2 might be SPRY4-IT1 novel targets in CCA cells. In addition, correlation analysis showed that there is an inverse relationship between SPRY4-IT1 and KLF2 or LATS2 expression in CCA tissues (Additional file 5: Figure S2C and D). Afterwards, silencing of LSD1 and EZH2 enhanced LATS2 expression, while knockdown of DNMT1 and EZH2 upregulated KLF2 expression in both HuCCT1 and RBE cells. However, RND1 expression was not affected by LSD1, EZH2 or DNMT1 (Fig. 5e and Additional file 5: Figure S2E-G). We next performed ChIP assays to examine the regulatory mechanisms. The data revealed that EZH2 could directly bind to LATS2 and KLF2 promoter region and mediate H3K27 trimethylation; DNMT1 could directly bind to the KLF2 promoter region; LSD1 directly binds to the LATS2 promoter region and induced H3K4 demethylation modification. In addition, silencing the transcription of SPRY4-IT1 decreased their binding ability and induced modification in HuCCT1 and RBE cells (Fig. 5f).

### SPRY4-IT1 functions as a ceRNA and sponges miR-101-3p in CCA cells

Interestingly, previous publication reported that SPRY4-IT1 positively regulates posttranscriptional expression of EZH2 by sponging miR-101-3p in bladder cancer [18]. In the present study, our findings showed that the expression of miR-101-3p is strongly affected by SPRY4-IT1 expression in CCA cells (Additional file 6: Figure S3A and B). EZH2 was also downregulated after SPRY4-IT1 knockdown or miR-101-3p overexpression (Additional file 6: Figure S3C and D). Then, we mutated the miR-101-3p binding site in SPRY4-IT1 by site-directed mutagenesis. As expected, the miR-101-3p mediated suppression of luciferase activity was abolished in this mutated SPRY4-IT1 construct compared with the wild-type vector (Additional file 6: Figure S3E). In addition, EZH2 was identified as a target of miR-101-3p by luciferase reporter assay (Additional file 6: Figure S3F). Furthermore, increased expression of miR-101-3p impaired CCA cell proliferation and invasive capabilities (Additional file 6: Figure S3G and H). Collectively, these results demonstrate that SPRY4-IT1 functions as a ceRNA for miR-101-3p, thereby derepresses EZH2 expression in CCA.

### SPRY4-IT1 oncogenic properties are partly dependent on repressing KLF2 and LATS2 expression

**Fig. 6:**
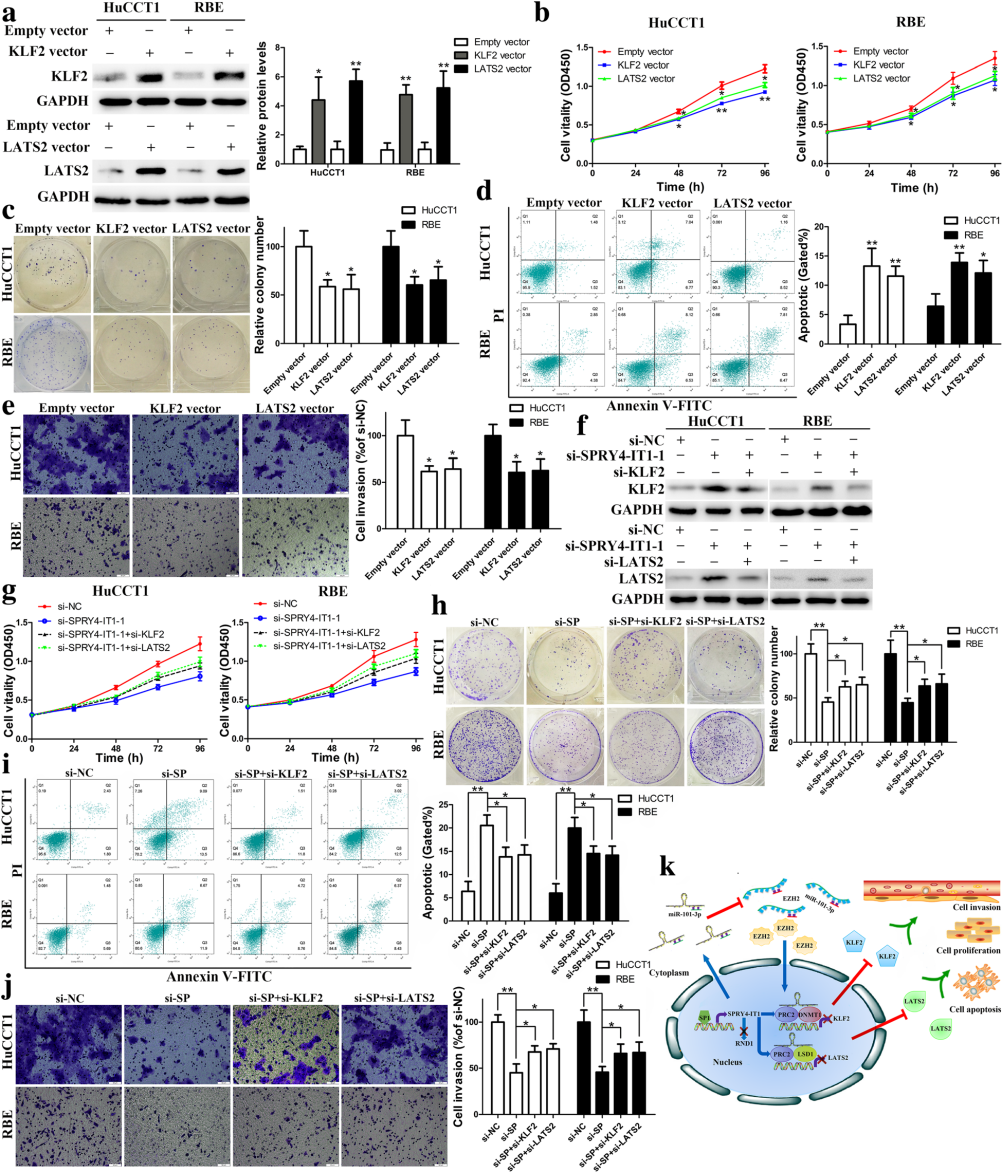
SPRY4-IT1 promotes CCA cell proliferation and invasion in part by regulating KLF2 and LATS2 expression. **a** Western blot assays were used to determine the expression of KLF2 and LATS2 in HuCCT1 and RBE cells. **b**-**e** CCK-8, colony formation, flow cytometric and transwell assays were used to determine the viability, colony forming ability, apoptosis and invasion of HuCCT1 and RBE cells. **f** Western blot assays were used to determine the expression of KLF2 and LATS2 in HuCCT1 and RBE cells. **g**-**j** CCK-8, colony formation, flow cytometric and transwell assays were used to determine the viability, colony forming ability, apoptosis and invasion of HuCCT1 and RBE cells. **k** Summary of the regulation and mechanism of SPRY4-IT1 in CCA cells. *P < 0.05, **P < 0.01

To address whether KLF2 and LATS2 are associated with SPRY4-IT1-mediated increases in cell growth and invasion, we upregulated KLF2 and LATS2 expression in CCA cells (Fig. 6a). As a result, overexpressed KLF2 or LATS2 significantly inhibited CCA cell proliferation according to the CCK-8 and colony formation assays (Fig. 6b and c). Moreover, flow cytometry analyses for apoptosis demonstrated that the groups with increased KLF2 or LATS2 expression had more apoptotic cells (Fig. 6d). Cell invasive potential was also inhibited after KLF2 or LATS2 increased (Fig. 6e). These findings imply that KLF2 and LATS2 may play a key role in CCA cell proliferation, apoptosis and invasion. Further rescue assays were performed to determine whether SPRY4-IT1 regulates CCA oncogenic properties partly by suppressing KLF2 and LATS2 expression. CCA cells were co-transfected with si-SPRY4-IT1–1 and si-KLF2; this co-transfection inhibited the increased KLF2 expression induced by SPRY4-IT1 knockdown. Similar results were found in si-SPRY4-IT1-LATS2 co-transfection groups (Fig. 6f). CCK-8 and colony formation assays showed that co-transfection could partially rescue si-SPRY4-IT1-impaired proliferation and clonogenic capacities in HuCCT1 and RBE cells (Fig. 6g and h). Additionally, similar results were found for cell apoptosis (Fig. 6i). Furthermore, transwell assays showed that inhibition of KLF2 and LATS2 partly rescued the SPRY4-IT1 downregulation-induced invasion decrease in CCA cells (Fig. 6j). These data indicate that SPRY4-IT1 promotes tumor cell aggressiveness partly via downregulating KLF2 and LATS2 expression in CCA.

## Discussion

Currently, radical operation and chemoradiotherapy, which are comprehensive treatments, have become the core approaches for treating CCA. Although these types of treatment can prolong lifespan and alleviate patient suffering, the prognosis for CCA remains poor [23, 24]. Recently, lncRNAs have emerged as crucial regulators of gene expression and key players in multiple cancers [25]. For example, increased levels of GAS5 can inhibit gastric cancer cell proliferation and induce apoptosis both in vitro and in vivo [26]. In addition, a number of lncRNAs, including MALAT1 and AFAP1-AS1, have been reported to be involved in CCA cell growth and metastasis [27–29]. In the current study, we focused on a novel cancer-associated lncRNA, SPRY4-IT1, whose expression levels are distinctly higher in CCA tissues than non-tumorous tissues. Aberrant expression levels of SPRY4-IT1 were correlated with tumor stage and TNM stage. Besides, SPRY4-IT1 could serve as an independent prognostic indicator for CCA patients proved by multivariate Cox regression analyses. The findings above suggest that SPRY4-IT1 may function as an oncogene and play a critical role in CCA development and progression. On the contrary, SPRY4-IT1 seems down-regulated in non-small-cell lung cancer and acts as a potential tumor suppressor [17], which suggests that SPRY4-IT1 may exert an oncogene or tumor suppressor function depending on the tissue-specific expression pattern and circumstances.

Recently, emerging evidence indicated that the expression of lncRNAs can be regulated by transcription factors just like some protein coding genes. For example, transcription factor E2F1 activates lncRNA HOXA11-AS transcription in gastric cancer [30]. In the present study, SPRY4-IT1 upregulation in CCA could be activated by SP1. Subsequent in vitro and in vivo studies indicated that SPRY4-IT1 silencing significantly inhibited malignant biological phenotypes of CCA cells. SPRY4-IT1 protected against cell apoptosis partly through Bcl-2/caspase-3 pathway. EMT, a major mechanism involved in tumor metastasis, causes the loss of cell-cell adhesion and increases migration and invasion capabilities [31]. E-cadherin is directly suppressed by Snail, which in turn induces EMT in epithelial tumor cells [32]. Here, we found a decrease in migration and invasion potentials after SPRY4-IT1 was silenced partly by reversing Snail mediated EMT. Herein, the SPRY4-IT1/Snail/E-cadherin axis may play a critical role in facilitating CCA metastatic properties.

After validating the role of SPRY4-IT1 as an oncogene in CCA, we explored the molecular mechanisms underlying the altered malignant phenotypes. Currently, several lncRNAs have been well characterized; these lncRNAs can modulate gene expression via various mechanisms, including chromatin modification, transcription and post-transcriptional processing. Recently, evidence has demonstrated that several lncRNAs regulate downstream effectors via interacting with RNA binding proteins such as EZH2, SUZ12, LSD1, STAU1, DNMT1 et al. [33, 34]. Previous publication has reported that EZH2-driven H3K27 methylation and LSD1 mediated H3K4 demethylation at the transcriptional level can be regulated by lncRNAs [35]. In our study, there is a direct interaction between SPRY4-IT1 and EZH2, LSD1 and DNMT1 in HuCCT1 and RBE cells. Further RT-qPCR analysis and correlation analysis showed that RND1, KLF2 and LATS2 may be novel targets of SPRY4-IT1 in CCA. However, silencing of EZH2, LSD1 or DNMT1 did not affect the expression of RND1 but enhanced KLF2 and LATS2 expression in HuCCT1 and RBE cells. The fact above suggested that SPRY4-IT1 may regulate RND1 through other mechanisms. Importantly, ChIP assays determined that SPRY4-IT1 could recruit EZH2 and DNMT1 to KLF2 promoter region and recruit EZH2 and LSD1 to LATS2 promoter region, respectively, and inhibit their transcription. KLF2, a tumor suppressor and member of the KLF family, contains Cys2/His2 zinc finger motifs and acts as transcription factors to regulate downstream targets including cyclin-dependent kinase genes [36]. Its expression is suppressed in various carcinomas, such as non-small cell lung cancer and pancreatic ductal adenocarcinoma [37, 38]. LATS2 is a key member of the Hippo pathway and functions as a tumor suppressor owing to its role in the suppression of cell growth and silencing the transcriptional co-activators YAP and TAZ [39]. It is frequently reduced in malignancies such as breast cancer and mesothelioma [40, 41]. In the current study, our findings showed that KLF2 and LATS2 functioned as a tumor suppressor in CCA. Importantly, rescue experiments determined that the oncogenic function of SPRY4-IT1 is partly depends on repressing KLF2 and LATS2 expression. These findings implied that SPRY4-IT1 may indirectly influence downstream targets of KLF2 and LATS2 such as cyclin-dependent kinase and Hippo pathway to exert oncogenic properties in CCA. In our study, SPRY4-IT1 RNAs were also located in the cytoplasm, which made us to investigate whether SPRY4-IT1 may play a role at post-transcriptional level in CCA. As we expected, SPRY4-IT1 acts as a ceRNA for miR-101-3p in CCA, and binding with miR-101-3p releases its inhibition of EZH2 mRNA, resulting in elevated EZH2 protein levels. EZH2 could then enter into the nucleus, which could further be recruited by SPRY4-IT1 to repress KLF2 and LATS2 transcription (Fig. 6k). In the present study, other possible targets and mechanisms underlying the regulatory actions were not explored sufficiently and a larger cohort of patients should be recruited to further validate the clinical value of SPRY4-IT1.

## Conclusions

In summary, our study first revealed that SPRY4-IT1 is upregulated in human CCA tissues and cells, and its overexpression may be an unfavorable prognostic factor for patients with CCA. SP1 could bind directly to the SPRY4-IT1 promoter region and activate its transcription. In addition, SPRY4-IT1 silencing yielded tumor suppressive effects both in vitro and in vivo. Importantly, the oncogenic effects of SPRY4-IT1 may occur via its binding to EZH2, LSD1 and DNMT1 to induce the epigenetic silencing of KLF2 and LATS2 transcription. SPRY4-IT1 also functioned as a molecular sponge for miR-101-3p, antagonizing its ability to repress EZH2 protein translation. Ultimately, these results may expedite the appraisal of novel predictive or therapeutic targets for CCA.

## Additional files

Additional file 1: **Table S1.** Primer sequences and siRNAs/shRNA oligonucleotides information. (XLSX 12 kb)

Additional file 2: **Table S2.** Relationship between SPRY4-IT1 expression and clinicopathologic features of CCA patients. (DOCX 15 kb)

Additional file 3 : **Table S3.** Univariate and multivariate Cox regression analysis of PFS and OS of CCA patients in study cohort. (DOCX 17 kb)

Additional file 4: **Figure S1.** **A** Prediction of SP1 binding sites in SPRY4-IT1 promoter region using JASPAR. **B** RT-qPCR was performed to measure the expression of SP1 after transfection. **P < 0.01. (TIFF 488 kb)

Additional file 5: **Figure S2.** **A** The interaction of SPRY4-IT1 and potential RNA binding proteins was predicted on http://pridb.gdcb.iastate.edu/RPISeq/. **B** U1 binding with SNRNP70 was used as a positive control in RIP assays using HuCCT1 and RBE cell extracts. **C-D** Association analysis of the relationship between SPRY4-IT1 and KLF2 /LATS2 expression levels in 20 paired CCA tissues. **E-G** EZH2, LSD1 and DNMT1 levels were detected in cells transfected with EZH2, LSD1 or DNMT1 siRNA or si-NC. **P < 0.01. (TIFF 770 kb)

Additional file 6: **Figure S3.** **A** MiR-101-3p levels were examined in HuCCT1 and RBE cells after transfected with si-SPRY4-IT1–1 or si-NC. **B** MiR-101-3p levels were examined in HuCCT1 and RBE cells after SPRY4-IT1 overexpression. **C** MiR-101-3p levels were examined in HuCCT1 and RBE cells after transfected with miR-101-3p mimics or miR-NC. **D** EZH2 protein levels were examined in HuCCT1 and RBE cells transfected with si-SPRY4-IT1–1, si-NC, miR-101-3p mimics or miR-NC by Western blotting. **E** Luciferase reporter assays were used to determine the interacting activity between miR-101-3p and SPRY4-IT1. **F** Luciferase reporter assays were used to determine the interacting activity between miR-101-3p and 3’UTR of EZH2. **G** Proliferation curves were determined in HuCCT1 and RBE cells after transfected with miR-101-3p mimics or miR-NC by CCK-8 assays. **H** Cell invasive capacities were examined in HuCCT1 and RBE cells after transfected with miR-101-3p mimics or miR-NC by transwell assays. *P < 0.05, **P < 0.01. (TIFF 3615 kb)
