# Supplementary material for: SP1-induced upregulation of lncRNA SPRY4-IT1 exerts oncogenic properties by scaffolding EZH2/LSD1/DNMT1 and sponging miR-101-3p in cholangiocarcinoma
Source: J Exp Clin Cancer Res. 2018 Apr 11;37:81. doi: 10.1186/s13046-018-0747-x (PMC5896100; doi:10.1186/s13046-018-0747-x)
Supplement: Supplementary file 2 — Table S2. Relationship between SPRY4-IT1 expression and clinicopathologic features of CCA patients. (DOCX 15 kb) [file 13046_2018_747_MOESM2_ESM.docx]

**Additional file 2: Table S2** Relationship between SPRY4-IT1 expression and clinicopathologic features of CCA patients (n=70)

| Clinicopathologic | No. of patients | SPRY4-IT1 expression | | *P*-value |
| --- | --- | --- | --- | --- |
| features | (n) | High (n) | Low (n) |  |
| Gender |  |  |  |  |
| Male | 31 | 20 | 11 | 0.466 |
| Female | 39 | 21 | 18 |  |
| Age |  |  |  |  |
| ＜60 | 41 | 25 | 16 | 0.806 |
| ≥60 | 29 | 16 | 13 |  |
| Tumor site |  |  |  |  |
| Intrahepatic | 12 | 6 | 6 | 0.536 |
| Extrahepatic | 58 | 35 | 23 |  |
| Tumor stage |  |  |  |  |
| T1-2 | 28 | 11 | 17 | 0.013 |
| T3-4 | 42 | 30 | 12 |  |
| Lymph node invasion |  |  |  |  |
| Present | 38 | 24 | 14 | 0.469 |
| Absent | 32 | 17 | 15 |  |
| TNM stage |  |  |  |  |
| I-II | 23 | 9 | 14 | 0.038 |
| III-IV | 47 | 32 | 15 |  |
| Differentiation grade |  |  |  |  |
| Well/moderately | 25 | 13 | 12 | 0.454 |
| Poorly/undifferentiated | 45 | 28 | 17 |  |
