# Supplementary material for: SP1-induced upregulation of lncRNA SPRY4-IT1 exerts oncogenic properties by scaffolding EZH2/LSD1/DNMT1 and sponging miR-101-3p in cholangiocarcinoma
Source: J Exp Clin Cancer Res. 2018 Apr 11;37:81. doi: 10.1186/s13046-018-0747-x (PMC5896100; doi:10.1186/s13046-018-0747-x)
Supplement: Supplementary file 3 — : Table S3. Univariate and multivariate Cox regression analysis of PFS and OS of CCA patients in study cohort. (DOCX 17 kb) [file 13046_2018_747_MOESM3_ESM.docx]

**Additional file 3: Table S3** Univariate and multivariate Cox regression analysis of PFS and OS of CCA patients in study cohort (n=70)

| Variables | PFS | | | OS | | |
| --- | --- | --- | --- | --- | --- | --- |
|  | HR | 95% CI | *P*-value | HR | 95% CI | *P*-value |
| Univariate analysis | | | | | | |
| Gender (Male vs. Female) | 1.108 | 0.668-1.838 | 0.692 | 1.142 | 0.686-1.901 | 0.609 |
| Age (≥60 vs. ＜60) | 0.906 | 0.544-1.510 | 0.705 | 0.865 | 0.516-1.451 | 0.584 |
| Tumor site  (Extrahepatic vs. Intrahepatic) | 0.766 | 0.398-1.475 | 0.425 | 0.744 | 0.386-1.432 | 0.376 |
| Tumor stage (T3-4 vs. T1-2) | 1.851 | 1.090-3.144 | 0.023 | 1.741 | 1.023-2.962 | 0.041 |
| Lymph node invasion  (Positive vs. Negative) | 1.544 | 0.922-2.585 | 0.099 | 1.561 | 0.929-2.624 | 0.093 |
| TNM stage (III-IV vs. I-II) | 2.629 | 1.482-4.666 | 0.001 | 2.633 | 1.470-4.717 | 0.001 |
| Differentiation grade (Poorly/undifferentiated vs. Well/moderately) | 1.415 | 0.826-2.423 | 0.206 | 1.447 | 0.838-2.499 | 0.185 |
| SPRY4-IT1 expression  (High vs. Low) | 2.311 | 1.335-3.999 | 0.003 | 2.199 | 1.276-3.788 | 0.005 |
| Multivariate analysis |  |  |  |  |  |  |
| Tumor stage (T3-4 vs. T1-2) |  |  | 0.300 |  |  | 0.350 |
| TNM stage (III-IV vs. I-II) | 2.309 | 1.282-4.157 | 0.005 | 2.329 | 1.283-4.228 | 0.005 |
| SPRY4-IT1 expression  (High vs. Low) | 1.970 | 1.122-3.461 | 0.018 | 1.876 | 1.073-3.280 | 0.027 |
