# Supplementary figures and images for: SP1-induced upregulation of lncRNA SPRY4-IT1 exerts oncogenic properties by scaffolding EZH2/LSD1/DNMT1 and sponging miR-101-3p in cholangiocarcinoma
Source: J Exp Clin Cancer Res. 2018 Apr 11;37:81. doi: 10.1186/s13046-018-0747-x (PMC5896100; doi:10.1186/s13046-018-0747-x)

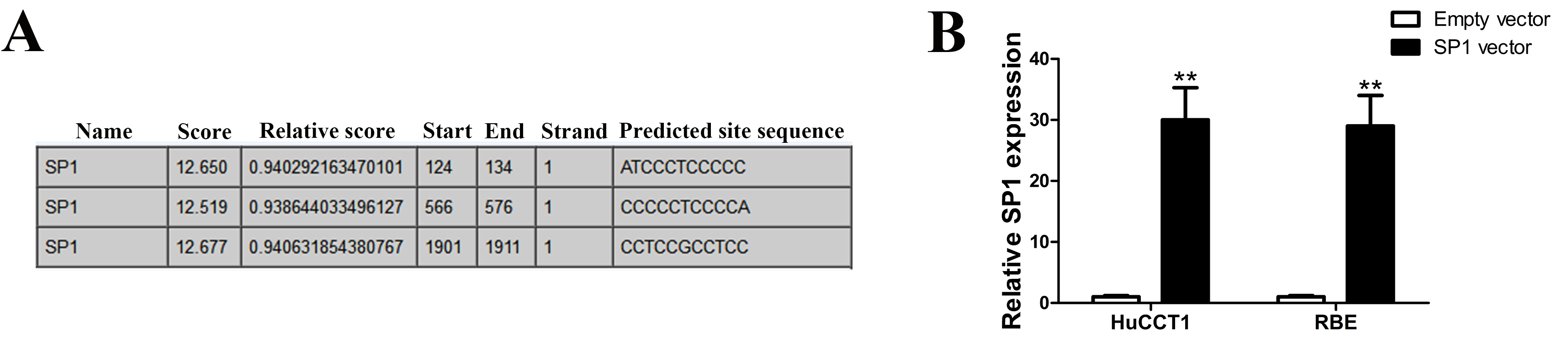

Supplement: Supplementary file 4 — Figure S1. A Prediction of SP1 binding sites in SPRY4-IT1 promoter region using JASPAR. B RT-qPCR was performed to measure the expression of SP1 after transfection. **P < 0.01. (TIFF 488 kb) [file 13046_2018_747_MOESM4_ESM.tif]

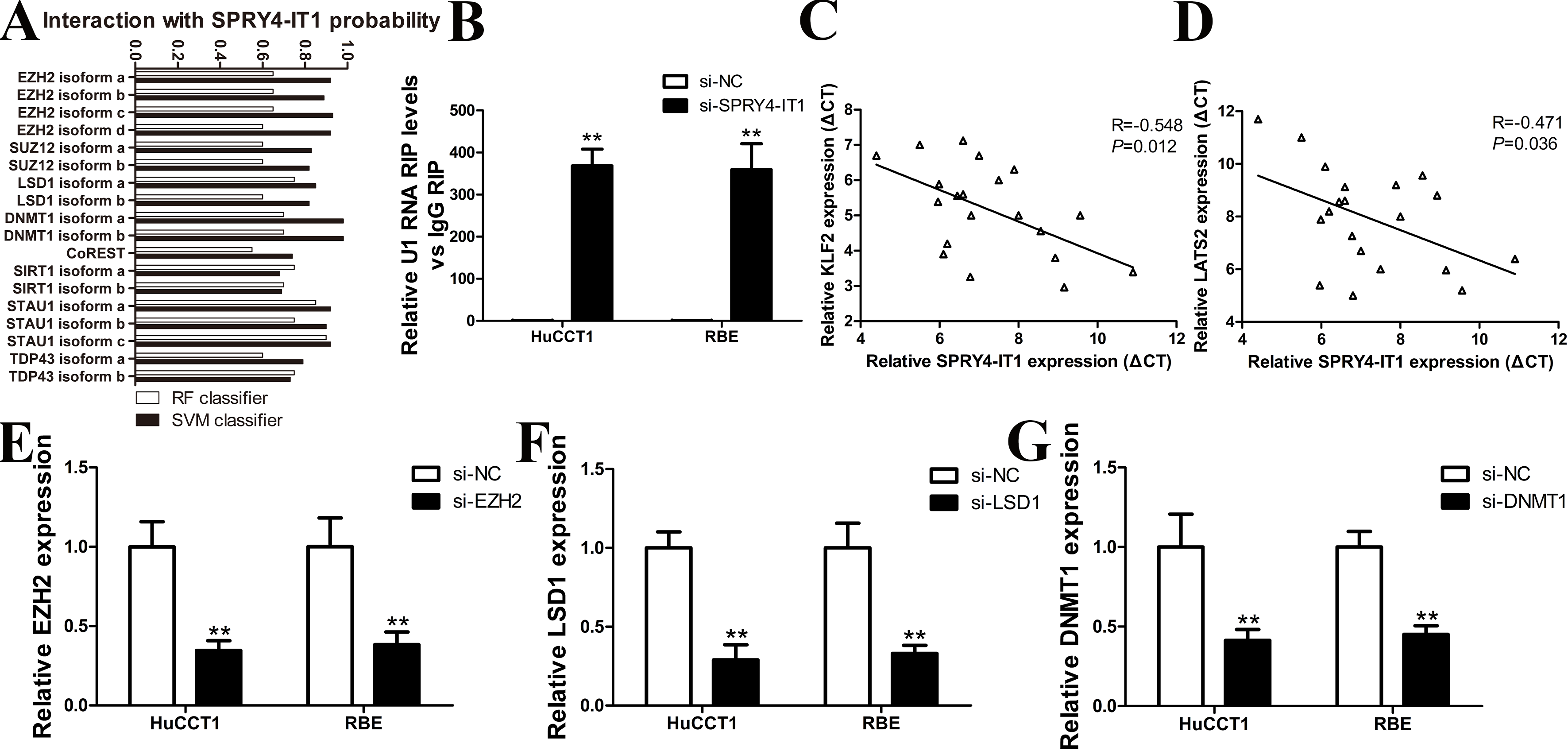

Supplement: Supplementary file 5 — Figure S2. A The interaction of SPRY4-IT1 and potential RNA binding proteins was predicted on http://pridb.gdcb.iastate.edu/RPISeq/. B U1 binding with SNRNP70 was used as a positive control in RIP assays using HuCCT1 and RBE cell extracts. C-D Association analysis of the relationship between SPRY4-IT1 and KLF2 /LATS2 expression levels in 20 paired CCA tissues. E-G EZH2, LSD1 and DNMT1 levels were detected in cells transfected with EZH2, LSD1 or DNMT1 siRNA or si-NC. **P < 0.01. (TIFF 770 kb) [file 13046_2018_747_MOESM5_ESM.tif]

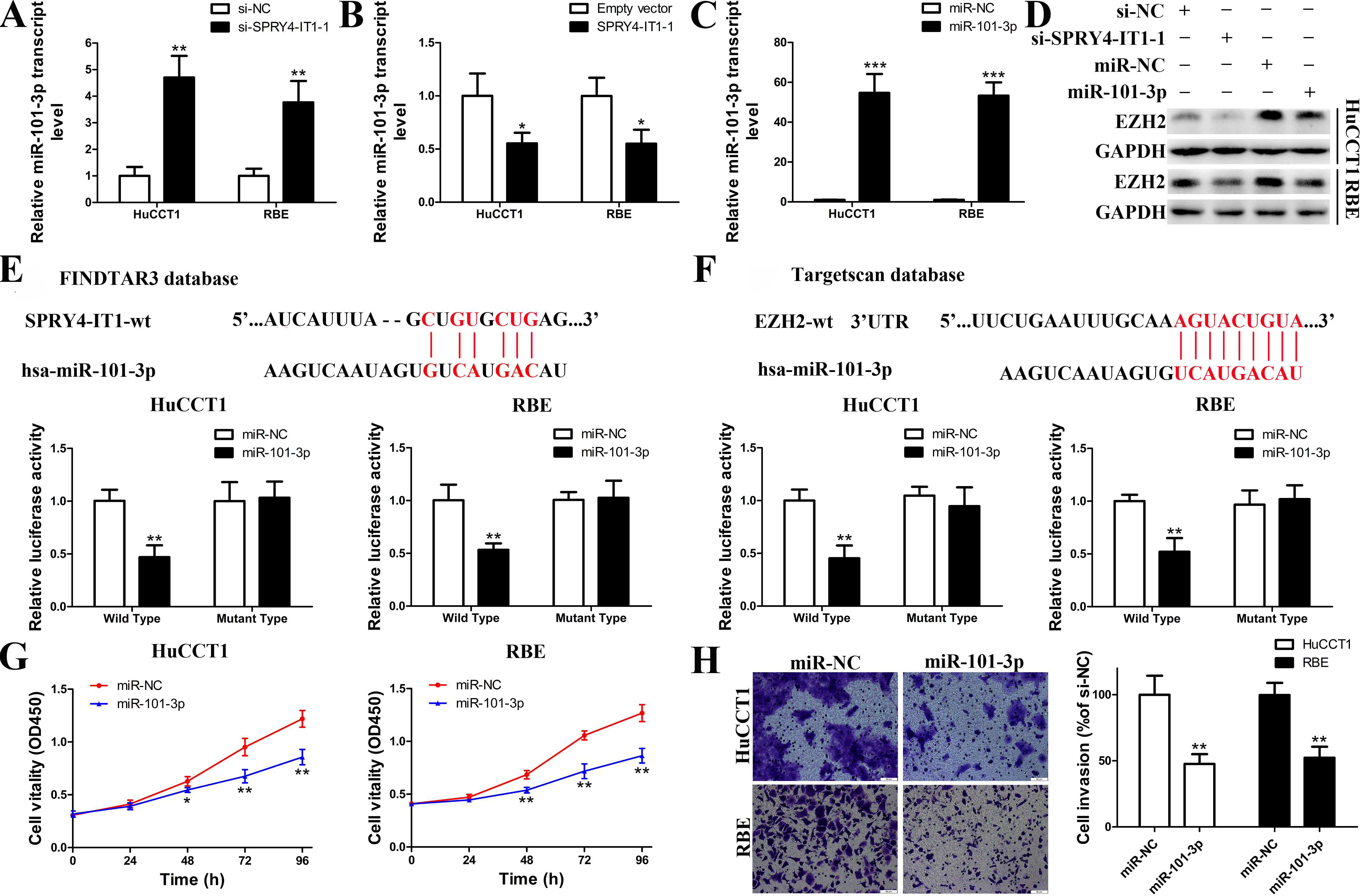

Supplement: Supplementary file 6 — Figure S3. A MiR-101-3p levels were examined in HuCCT1 and RBE cells after transfected with si-SPRY4-IT1–1 or si-NC. B MiR-101-3p levels were examined in HuCCT1 and RBE cells after SPRY4-IT1 overexpression. C MiR-101-3p levels were examined in HuCCT1 and RBE cells after transfected with miR-101-3p mimics or miR-NC. D EZH2 protein levels were examined in HuCCT1 and RBE cells transfected with si-SPRY4-IT1–1, si-NC, miR-101-3p mimics or miR-NC by Western blotting. E Luciferase reporter assays were used to determine the interacting activity between miR-101-3p and SPRY4-IT1. F Luciferase reporter assays were used to determine the interacting activity between miR-101-3p and 3’UTR of EZH2. G Proliferation curves were determined in HuCCT1 and RBE cells after transfected with miR-101-3p mimics or miR-NC by CCK-8 assays. H Cell invasive capacities were examined in HuCCT1 and RBE cells after transfected with miR-101-3p mimics or miR-NC by transwell assays. *P < 0.05, **P < 0.01. (TIFF 3615 kb) [file 13046_2018_747_MOESM6_ESM.tif]
